# 9-(4-Bromo­but­yl)-9*H*-carbazole

**DOI:** 10.1107/S1600536812022398

**Published:** 2012-05-23

**Authors:** Rodolfo Moreno-Fuquen, Carlos Grande, Rigoberto C. Advincula, Juan C. Tenorio, Javier Ellena

**Affiliations:** a Departamento de Química, Facultad de Ciencias, Universidad del Valle, Apartado 25360, Santiago de Cali, Colombia; b Programa de Ingenieria Agroindustrial, Universidad San Buenaventura, AA 7154, Santiago de Cali, Colombia; c Case Western Reserve University, Department of Macromolecular Science and Engineering, 2100 Adelbert Road, Kent Hale Smith Bldg, Cleveland, Ohio 44106, USA; d Instituto de Física de São Carlos, IFSC, Universidade de São Paulo, USP, São Carlos, SP, Brazil

## Abstract

In the title compound, C_16_H_16_BrN, the tricyclic carbazole system is essentially planar (r.m.s. deviation of all non-H atoms = 0.010 Å). The dihedral angle between the two outer carbazole rings is 1.1 (3)°. There are no directional inter­molecular contacts in the crystal packing.

## Related literature

For synthesis and properties of carbazole derivatives, see: Bo *et al.* (1998 ▶). For chemical properties of carbazoles, see: Knolker & Reddy (2002 ▶), for their physical properties, see: Koyuncu *et al.* (2011 ▶), for their medicinal properties, see: Zhang *et al.* (2010 ▶) and for their opto-electronic and electrochemical properties, see: Taranekar *et al.* (2007 ▶); Morisaki *et al.* (2009 ▶). For related structures, see: Gerkin & Reppart (1986 ▶); Duan *et al.* (2005 ▶); Zhou *et al.* (2008 ▶); Panchatcharam *et al.* (2011 ▶).
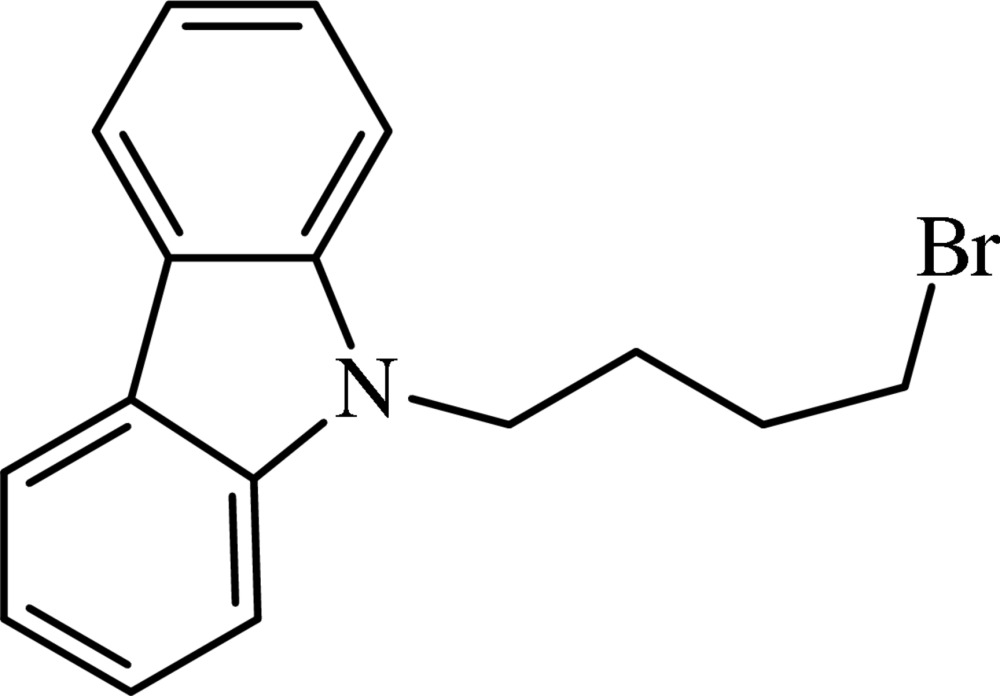


## Experimental

### 

#### Crystal data

C_16_H_16_BrN
*M* *_r_* = 302.21
Orthorhombic, 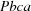

*a* = 16.0949 (4) Å
*b* = 7.7012 (2) Å
*c* = 22.6874 (4) Å
*V* = 2812.10 (11) Å^3^
*Z* = 8
Mo *K*α radiation
μ = 2.91 mm^−1^
*T* = 293 K
0.24 × 0.21 × 0.17 mm

#### Data collection

Nonius KappaCCD diffractometer
Absorption correction: multi-scan (*SADABS*; Sheldrick, 1996 ▶) *T*_min_ = 0.510, *T*_max_ = 0.589
25173 measured reflections
2860 independent reflections
2002 reflections with *I* > 2σ(*I*)
*R*_int_ = 0.042

#### Refinement

*R*[*F* ^2^ > 2σ(*F* ^2^)] = 0.049
*wR*(*F* ^2^) = 0.146
*S* = 1.03
2860 reflections
163 parameters
H-atom parameters constrained
Δρ_max_ = 0.38 e Å^−3^
Δρ_min_ = −0.45 e Å^−3^

### 

Data collection: *COLLECT* (Nonius, 2000 ▶); cell refinement: *SCALEPACK* (Otwinowski & Minor, 1997 ▶); data reduction: *DENZO* (Otwinowski & Minor, 1997 ▶) and *SCALEPACK*; program(s) used to solve structure: *SHELXS97* (Sheldrick, 2008 ▶); program(s) used to refine structure: *SHELXL97* (Sheldrick, 2008 ▶); molecular graphics: *ORTEP-3 for Windows* (Farrugia, 1997 ▶) and *Mercury* (Macrae *et al.*, 2006 ▶); software used to prepare material for publication: *WinGX* (Farrugia, 1999 ▶).

## Supplementary Material

Crystal structure: contains datablock(s) I, global. DOI: 10.1107/S1600536812022398/zs2201sup1.cif

Structure factors: contains datablock(s) I. DOI: 10.1107/S1600536812022398/zs2201Isup2.hkl

Supplementary material file. DOI: 10.1107/S1600536812022398/zs2201Isup3.cml

Additional supplementary materials:  crystallographic information; 3D view; checkCIF report

## Figures and Tables

**Fig. 1.**
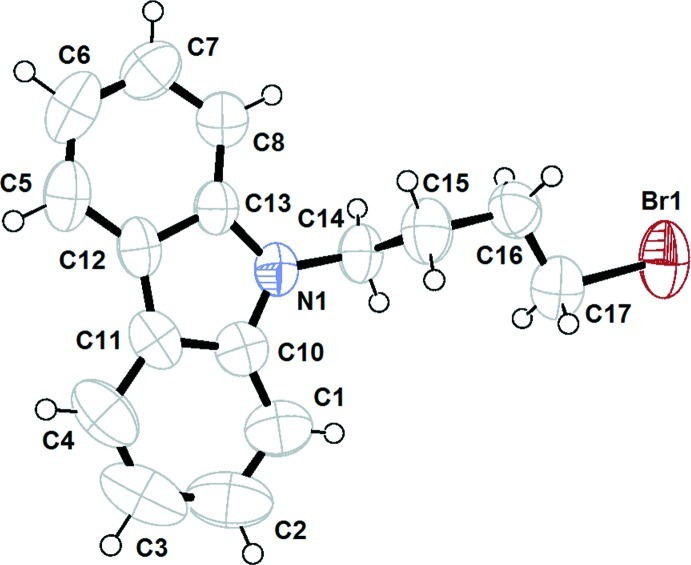
Molecular conformation and atom numbering scheme for the title compound with displacement ellipsoids drawn at the 50% probability level. H atoms are shown as spheres of arbitrary radius.

## supplementary crystallographic information

### Comment

The carbazole ring has a highly conjugated π system with desirable optical and charge-transport properties. These characteristics make it an excellent candidate for applications in different areas of science. Indeed, carbazole and its derivatives, heterocyclic compounds with a N atom in their structure, have interesting chemical (Knolker & Reddy, 2002), physical (Koyuncu *et al.*, 2011) and medicinal (Zhang *et al.*, 2010) properties. Polymers based on carbazole units become promising materials because of their optical, electronic and electrochemical behaviors (Taranekar *et al.*, 2007; Morisaki *et al.*, 2009). One of these derivatives, 9-(4-bromobutyl)-9*H*-carbazole, the title compound C_16_H_16_BrN, was synthesized and its structure is reported here.

In the title compound (Fig. 1) the tricyclic carbazole system is essentially planar (r.m.s. deviation of all non-hydrogen atoms = 0.0100 Å), with a dihedral angle of 1.1 (3)° between two outer rings of the molecule. Other related systems are similar (Gerkin & Reppart, 1986; Duan *et al.*, 2005). Bond distances and bond angles in the carbazole ring are comparable with those observed in similar structures (Zhou *et al.*, 2008; Panchatcharam *et al.*, 2011). The *n*-butylbromide chain adopts a semi-extended conformation, with torsion angles C10—N1—C14—C15, N1—C14—C15—C16, C14—C15—C16—C17 and C15—C16—C17—Br1 of -97.3 (4), -177.1 (3), -79.7 (4) and -178.8 (3)°, respectively. No Br···Br interactions or short intermolecular contacts are found in the crystal structure.

### Experimental

The synthesis of 9-(4-bromobutyl)-9*H*-carbazole was accomplished by a modified method as reported by Bo *et al.*, 1998. A mixture of 10.32 g (61.72 mmol) of carbazole in toluene (100 ml) containing 1,4-dibromobutane (118.2 g, 547.4 mmol) and tetrabutylammonium bromide (TBAB, 2.0 g) was stirred at 45 °C for 3 h. and then left overnight. After the aqueous layer was removed and washed three times with water and brine, the organic layer was dried over Na_2_SO_4_. The organic solvent was evaporated, and unreacted 1,4-dibromobutane was removed by vacuum distillation. The residue was recrystallized from ethanol to give 16.7 g (89.5% yield) of the title compound, m.p. 379 (1) K. _1_H NMR (400 MHz) δ, 8.10 (d, 2H, carbazole ring), 7.11–7.47 (m, 6H, carbazole ring), 4.35 (t, 2H, NCH_2_), 3.37(t, CH_2_Br), 1.91 (m, 4H, CH_2_CH_2_). IR (KBr): 3045 (Ar—H); 2939, 2925, 2855 (CH_2_); 1620, 1593 (carbazole ring).

### Refinement

All H-atoms were positioned geometrically using a riding model with C—H(aromatic) = 0.93 Å and C—H(methylene) = 0.97 Å and with *U*_iso_(H) = 1.2*U*_eq_(C).

### Crystal data

**Table:**

C_16_H_16_BrN	*D*_x_ = 1.428 Mg m^−^^3^
*M**_r_* = 302.21	Melting point: 379(1) K
Orthorhombic, *P**b**c**a*	Mo *K*α radiation, λ = 0.71073 Å
Hall symbol: -P 2ac 2ab	Cell parameters from 18997 reflections
*a* = 16.0949 (4) Å	θ = 2.9–26.4°
*b* = 7.7012 (2) Å	µ = 2.91 mm^−^^1^
*c* = 22.6874 (4) Å	*T* = 293 K
*V* = 2812.10 (11) Å^3^	Prism, colourless
*Z* = 8	0.24 × 0.21 × 0.17 mm
*F*(000) = 1232	

### Data collection

**Table:**

Nonius KappaCCD diffractometer	2860 independent reflections
Radiation source: fine-focus sealed tube	2002 reflections with *I* > 2σ(*I*)
Graphite monochromator	*R*_int_ = 0.042
CCD rotation images, thick slices scans	θ_max_ = 26.4°, θ_min_ = 3.6°
Absorption correction: multi-scan (*SADABS*; Sheldrick, 1996)	*h* = −20→20
*T*_min_ = 0.510, *T*_max_ = 0.589	*k* = −9→9
25173 measured reflections	*l* = −28→19

### Refinement

**Table:**

Refinement on *F*^2^	Primary atom site location: structure-invariant direct methods
Least-squares matrix: full	Secondary atom site location: difference Fourier map
*R*[*F*^2^ > 2σ(*F*^2^)] = 0.049	Hydrogen site location: inferred from neighbouring sites
*wR*(*F*^2^) = 0.146	H-atom parameters constrained
*S* = 1.03	*w* = 1/[σ^2^(*F*_o_^2^) + (0.064*P*)^2^ + 2.1492*P*] where *P* = (*F*_o_^2^ + 2*F*_c_^2^)/3
2860 reflections	(Δ/σ)_max_ < 0.001
163 parameters	Δρ_max_ = 0.38 e Å^−^^3^
0 restraints	Δρ_min_ = −0.45 e Å^−^^3^

### Special details

**Table:**

Geometry. All e.s.d.'s (except the e.s.d. in the dihedral angle between two l.s. planes) are estimated using the full covariance matrix. The cell e.s.d.'s are taken into account individually in the estimation of e.s.d.'s in distances, angles and torsion angles; correlations between e.s.d.'s in cell parameters are only used when they are defined by crystal symmetry. An approximate (isotropic) treatment of cell e.s.d.'s is used for estimating e.s.d.'s involving l.s. planes.
Refinement. Refinement of *F*^2^ against ALL reflections. The weighted *R*-factor *wR* and goodness of fit *S* are based on *F*^2^, conventional *R*-factors *R* are based on *F*, with *F* set to zero for negative *F*^2^. The threshold expression of *F*^2^ > σ(*F*^2^) is used only for calculating *R*-factors(gt) *etc*. and is not relevant to the choice of reflections for refinement. *R*-factors based on *F*^2^ are statistically about twice as large as those based on *F*, and *R*- factors based on ALL data will be even larger.

### Fractional atomic coordinates and isotropic or equivalent isotropic displacement parameters (Å^2^)

**Table:**

	*x*	*y*	*z*	*U*_iso_*/*U*_eq_	
Br	0.35086 (4)	1.17737 (6)	0.51126 (3)	0.1073 (3)	
N1	0.43223 (16)	0.4566 (3)	0.63370 (11)	0.0617 (6)	
C1	0.5664 (2)	0.4352 (6)	0.57887 (17)	0.0885 (11)	
H1	0.5559	0.5272	0.5534	0.106*	
C2	0.6389 (3)	0.3424 (9)	0.5758 (2)	0.1156 (19)	
H2	0.6783	0.3726	0.5476	0.139*	
C3	0.6552 (3)	0.2047 (9)	0.6134 (3)	0.118 (2)	
H3	0.7049	0.1442	0.6098	0.141*	
C4	0.5985 (3)	0.1559 (6)	0.6562 (2)	0.0960 (14)	
H4	0.6096	0.0633	0.6813	0.115*	
C5	0.4308 (3)	0.1312 (5)	0.74590 (18)	0.0816 (11)	
H5	0.4668	0.0449	0.7589	0.098*	
C6	0.3562 (3)	0.1551 (6)	0.7728 (2)	0.0954 (14)	
H6	0.3414	0.0846	0.8045	0.115*	
C7	0.3019 (3)	0.2824 (5)	0.75390 (18)	0.0859 (11)	
H7	0.2510	0.2951	0.7728	0.103*	
C8	0.3215 (2)	0.3911 (5)	0.70768 (16)	0.0702 (9)	
H8	0.2848	0.4770	0.6953	0.084*	
C10	0.5093 (2)	0.3865 (5)	0.62154 (14)	0.0655 (8)	
C11	0.5247 (2)	0.2484 (5)	0.66088 (16)	0.0681 (9)	
C12	0.4529 (2)	0.2368 (4)	0.69877 (14)	0.0624 (8)	
C13	0.39748 (19)	0.3677 (4)	0.68040 (14)	0.0578 (7)	
C14	0.3937 (2)	0.6011 (4)	0.60257 (15)	0.0690 (9)	
H14A	0.3347	0.5782	0.5985	0.083*	
H14B	0.4172	0.6086	0.5633	0.083*	
C15	0.4052 (3)	0.7739 (5)	0.63337 (18)	0.0841 (11)	
H15A	0.4641	0.8002	0.6356	0.101*	
H15B	0.3841	0.7652	0.6733	0.101*	
C16	0.3595 (2)	0.9255 (5)	0.60072 (19)	0.0807 (10)	
H16A	0.3055	0.8862	0.5872	0.097*	
H16B	0.3510	1.0218	0.6277	0.097*	
C17	0.4087 (3)	0.9825 (5)	0.55080 (19)	0.0903 (11)	
H17A	0.4160	0.8872	0.5233	0.108*	
H17B	0.4631	1.0198	0.5641	0.108*	

### Atomic displacement parameters (Å^2^)

**Table:**

	*U*^11^	*U*^22^	*U*^33^	*U*^12^	*U*^13^	*U*^23^
Br	0.1314 (5)	0.0737 (3)	0.1168 (4)	−0.0018 (2)	−0.0395 (3)	0.0214 (2)
N1	0.0734 (16)	0.0522 (14)	0.0593 (15)	0.0028 (12)	−0.0059 (12)	0.0041 (12)
C1	0.086 (2)	0.113 (3)	0.066 (2)	−0.008 (2)	0.002 (2)	−0.015 (2)
C2	0.083 (3)	0.180 (6)	0.084 (3)	0.001 (3)	0.003 (2)	−0.043 (4)
C3	0.081 (3)	0.167 (6)	0.106 (4)	0.039 (3)	−0.023 (3)	−0.062 (4)
C4	0.090 (3)	0.102 (3)	0.096 (3)	0.032 (2)	−0.035 (3)	−0.031 (2)
C5	0.101 (3)	0.063 (2)	0.081 (2)	−0.009 (2)	−0.031 (2)	0.0146 (18)
C6	0.115 (4)	0.088 (3)	0.083 (3)	−0.033 (3)	−0.014 (3)	0.025 (2)
C7	0.081 (3)	0.095 (3)	0.081 (2)	−0.023 (2)	0.000 (2)	0.006 (2)
C8	0.0662 (19)	0.069 (2)	0.075 (2)	−0.0044 (17)	−0.0103 (17)	0.0006 (18)
C10	0.072 (2)	0.0667 (19)	0.0577 (18)	−0.0047 (16)	−0.0081 (16)	−0.0118 (16)
C11	0.070 (2)	0.0630 (19)	0.071 (2)	0.0079 (17)	−0.0221 (17)	−0.0164 (17)
C12	0.075 (2)	0.0460 (15)	0.0662 (19)	−0.0012 (15)	−0.0232 (16)	−0.0021 (14)
C13	0.0655 (18)	0.0486 (15)	0.0595 (17)	−0.0051 (14)	−0.0124 (15)	−0.0006 (13)
C14	0.086 (2)	0.0568 (18)	0.0639 (19)	−0.0002 (17)	−0.0141 (17)	0.0060 (15)
C15	0.106 (3)	0.066 (2)	0.080 (2)	−0.0028 (19)	−0.013 (2)	−0.0008 (18)
C16	0.079 (2)	0.067 (2)	0.096 (3)	−0.0004 (17)	0.0006 (19)	−0.0061 (19)
C17	0.098 (3)	0.077 (2)	0.095 (3)	−0.003 (2)	−0.010 (2)	0.008 (2)

### Geometric parameters (Å, º)

**Table:**

Br—C17	1.981 (4)	C7—C8	1.378 (5)
N1—C13	1.380 (4)	C7—H7	0.9300
N1—C10	1.380 (4)	C8—C13	1.383 (5)
N1—C14	1.457 (4)	C8—H8	0.9300
C1—C2	1.371 (7)	C10—C11	1.411 (5)
C1—C10	1.387 (5)	C11—C12	1.442 (5)
C1—H1	0.9300	C12—C13	1.409 (4)
C2—C3	1.385 (9)	C14—C15	1.514 (5)
C2—H2	0.9300	C14—H14A	0.9700
C3—C4	1.386 (8)	C14—H14B	0.9700
C3—H3	0.9300	C15—C16	1.567 (5)
C4—C11	1.389 (5)	C15—H15A	0.9700
C4—H4	0.9300	C15—H15B	0.9700
C5—C6	1.360 (6)	C16—C17	1.450 (6)
C5—C12	1.389 (5)	C16—H16A	0.9700
C5—H5	0.9300	C16—H16B	0.9700
C6—C7	1.382 (6)	C17—H17A	0.9700
C6—H6	0.9300	C17—H17B	0.9700
			
C13—N1—C10	108.9 (3)	C10—C11—C12	106.5 (3)
C13—N1—C14	125.4 (3)	C5—C12—C13	118.9 (4)
C10—N1—C14	125.8 (3)	C5—C12—C11	134.4 (3)
C2—C1—C10	117.3 (5)	C13—C12—C11	106.6 (3)
C2—C1—H1	121.3	N1—C13—C8	129.6 (3)
C10—C1—H1	121.3	N1—C13—C12	109.0 (3)
C1—C2—C3	121.9 (5)	C8—C13—C12	121.4 (3)
C1—C2—H2	119.0	N1—C14—C15	113.3 (3)
C3—C2—H2	119.0	N1—C14—H14A	108.9
C2—C3—C4	121.0 (4)	C15—C14—H14A	108.9
C2—C3—H3	119.5	N1—C14—H14B	108.9
C4—C3—H3	119.5	C15—C14—H14B	108.9
C3—C4—C11	118.5 (5)	H14A—C14—H14B	107.7
C3—C4—H4	120.7	C14—C15—C16	112.3 (3)
C11—C4—H4	120.7	C14—C15—H15A	109.1
C6—C5—C12	119.6 (4)	C16—C15—H15A	109.1
C6—C5—H5	120.2	C14—C15—H15B	109.1
C12—C5—H5	120.2	C16—C15—H15B	109.1
C5—C6—C7	120.9 (4)	H15A—C15—H15B	107.9
C5—C6—H6	119.5	C17—C16—C15	109.7 (3)
C7—C6—H6	119.5	C17—C16—H16A	109.7
C8—C7—C6	121.6 (4)	C15—C16—H16A	109.7
C8—C7—H7	119.2	C17—C16—H16B	109.7
C6—C7—H7	119.2	C15—C16—H16B	109.7
C7—C8—C13	117.6 (3)	H16A—C16—H16B	108.2
C7—C8—H8	121.2	C16—C17—Br	109.0 (3)
C13—C8—H8	121.2	C16—C17—H17A	109.9
N1—C10—C1	129.0 (4)	Br—C17—H17A	109.9
N1—C10—C11	109.0 (3)	C16—C17—H17B	109.9
C1—C10—C11	122.0 (4)	Br—C17—H17B	109.9
C4—C11—C10	119.3 (4)	H17A—C17—H17B	108.3
C4—C11—C12	134.3 (4)		
			
C10—C1—C2—C3	0.2 (7)	C4—C11—C12—C5	1.6 (7)
C1—C2—C3—C4	−0.5 (8)	C10—C11—C12—C5	−179.1 (4)
C2—C3—C4—C11	−0.1 (7)	C4—C11—C12—C13	−179.0 (4)
C12—C5—C6—C7	0.2 (6)	C10—C11—C12—C13	0.3 (3)
C5—C6—C7—C8	−0.7 (7)	C10—N1—C13—C8	−179.7 (3)
C6—C7—C8—C13	0.4 (6)	C14—N1—C13—C8	0.4 (5)
C13—N1—C10—C1	−179.4 (3)	C10—N1—C13—C12	−0.2 (3)
C14—N1—C10—C1	0.5 (5)	C14—N1—C13—C12	179.9 (3)
C13—N1—C10—C11	0.4 (3)	C7—C8—C13—N1	179.9 (3)
C14—N1—C10—C11	−179.7 (3)	C7—C8—C13—C12	0.5 (5)
C2—C1—C10—N1	−179.6 (4)	C5—C12—C13—N1	179.5 (3)
C2—C1—C10—C11	0.6 (5)	C11—C12—C13—N1	0.0 (3)
C3—C4—C11—C10	0.9 (5)	C5—C12—C13—C8	−1.0 (5)
C3—C4—C11—C12	−179.9 (4)	C11—C12—C13—C8	179.5 (3)
N1—C10—C11—C4	178.9 (3)	C13—N1—C14—C15	82.5 (4)
C1—C10—C11—C4	−1.2 (5)	C10—N1—C14—C15	−97.3 (4)
N1—C10—C11—C12	−0.4 (3)	N1—C14—C15—C16	−177.1 (3)
C1—C10—C11—C12	179.4 (3)	C14—C15—C16—C17	−79.7 (4)
C6—C5—C12—C13	0.6 (5)	C15—C16—C17—Br	−178.8 (3)
C6—C5—C12—C11	180.0 (4)		
